# Supplementary material for: pH Sensitive Dextran Coated Fluorescent Nanodiamonds as a Biomarker for HeLa Cells Endocytic Pathway and Increased Cellular Uptake
Source: Nanomaterials (Basel). 2021 Jul 15;11(7):1837. doi: 10.3390/nano11071837 (PMC8308332; doi:10.3390/nano11071837)
Supplement: Supplementary file 1 [file nanomaterials-11-01837-s001.zip › nanomaterials-1297921-supplementary.pdf]

nanomaterials

Supplementary Materials

pH Sensitive Dextran Coated Fluorescent Nanodiamonds as a Biomarker for HeLa Cells Endocytic Pathway and Increased Cellular Uptake

Linyan Nie †, Yue Zhang †, Lei Li, Patrick van Rijn and Romana Schirhagl *

Department of Biomedical Engineering, University Medical Center Groningen, University of Groningen, A. Deusinglaan 1, 9713 AV, Groningen, The Netherlands; niely0316@163.com (L.N.); yue.zhang@rug.nl (Y.Z.); l.li@umcg.nl (L.L.); p.van.rijn@umcg.nl (P.V.R.)
* Correspondence: romana.schirhagl@gmail.com
† These authors contributed equally to this work.

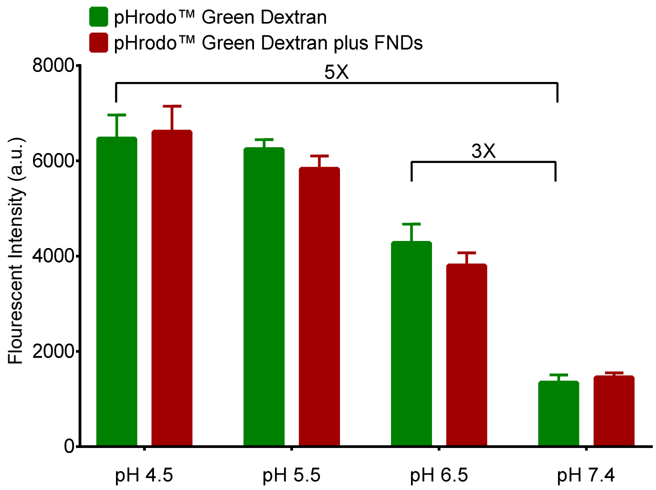

Figure S1. Measurement of fluorescent intensity changes in PBS at different pH values by a plate reader. These pH values were chosen according to match the inner acidic environment of endocytosis vehicles, pH = 4.5 in lysosomes, pH = 5.5 in late endosomes, pH = 6.5 in early endosomes, pH = 7.4 in cytoplasm or outer cell environment. Green colour stands for only pHrodo Green Dextran, red colour stands for pHrodo Green Dextran plus FNDs.

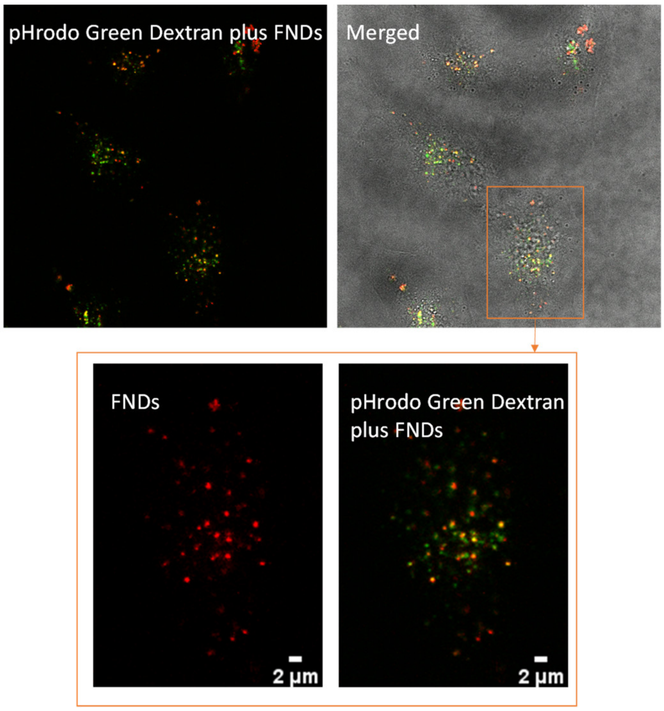

Figure S2. Cellular uptake of pHrodo Green Dextran adsorbed FNDs, incubation time is 0.5 + 3 hours, cells were incubated with pHrodo Green Dextran adsorbed FNDs for 0.5h and washed with PBS. Then PBS was replaced with fresh cell culture media, followed by 3 hrs incubation. Green: pHrodo Green Dextran, red: FNDs, scale bar is 2µm.

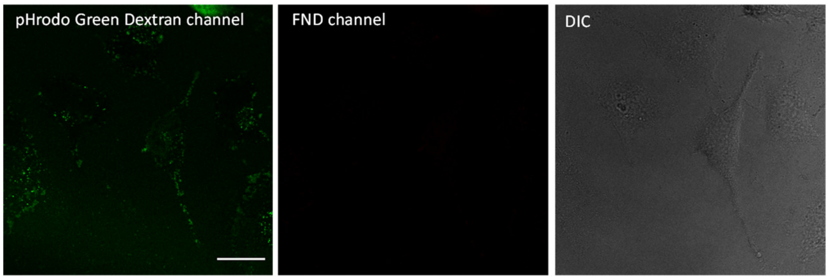

Figure S3. Control group; pHrodo Green Dextran incubated with cells for 0.5 hour. Green: pHrodo Green Dextran, scale bar is 20 µm.
